# Phenyllactic Acid Restores Intestinal Epithelial Barrier to Alleviate Hypertriglyceridemic Acute Pancreatitis via a PPARγ-Dependent Mechanism

**DOI:** 10.3390/antiox15060676

**Published:** 2026-05-28

**Authors:** Ze-Yun Cao, Xun Zou, Hong-Li Li, Xuan Kong, Li-Long Pan, Jun Yang, Xiao-Liang Dong

**Affiliations:** 1 MOE Medical Basic Research Innovation Center for Gut Microbiota and Chronic Diseases, Wuxi School of Medicine, Jiangnan University, Wuxi 214122, China; 6232803001@stu.jiangnan.edu.cn (Z.-Y.C.); 6232803015@stu.jiangnan.edu.cn (X.Z.); 6242803007@stu.jiangnan.edu.cn (H.-L.L.); 6242803006@stu.jiangnan.edu.cn (X.K.); llpan@jiangnan.edu.cn (L.-L.P.); 2 Affiliated Hospital of Jiangnan University, Jiangnan University, Wuxi 214122, China

**Keywords:** hypertriglyceridemia-associated acute pancreatitis, phenyllactic acid, intestinal barrier homeostasis, peroxisome proliferator-activated receptor gamma, oxidative stress

## Abstract

Hypertriglyceridemic acute pancreatitis (HTG-AP) progresses rapidly with poor prognosis. Intestinal barrier dysfunction and excessive oxidative stress contribute to its pathogenesis, but specific mediators linking gut injury, oxidative stress and pancreatic damage remain unclear. Here, we identify endogenous phenyllactic acid (PLA) as a critical metabolite regulating intestinal barrier integrity and oxidative homeostasis in HTG-AP. We noted serum PLA, a disease-associated metabolite whose reduction correlates with gut dysbiosis and pancreatic inflammation in HTG-AP. PLA supplementation in HTG-AP mice attenuated intestinal barrier dysfunction and mitigated intestinal oxidative stress, as evidenced by improved gut dysbiosis, reduced reactive oxygen species accumulation, restored superoxide dismutase activity, restored barrier integrity, reduced bacterial translocation to the pancreas, and decreased serum lipopolysaccharide levels, ultimately mitigating pancreatic injury. RNA sequencing of colonic tissue revealed peroxisome proliferator-activated receptor (PPAR) signaling as one of the most significantly altered pathways in HTG-AP. PPARγ expression was markedly reduced in colonic epithelial cells and upregulated upon PLA treatment. Knockdown of colonic epithelial PPARγ via adeno-associated virus abrogated the beneficial effects of PLA on intestinal barrier integrity, oxidative stress and pancreatic injury in HTG-AP mice. The protective effects of PLA were phenocopied by the PPARγ agonist rosiglitazone. Collectively, these findings identified gut microbiota-derived PLA as an endogenously derived metabolite modulating intestinal oxidative stress and barrier function. Using male C57BL/6J mice to establish an HTG-AP model, we further revealed that PLA exerts protective effects against HTG-AP by targeting colonic PPARγ to modulate the gut–pancreas axis, highlighting PLA as a promising candidate for targeted intervention in HTG-AP.

## 1. Introduction

Hypertriglyceridemic acute pancreatitis (HTG-AP) ranks as the third most common cause of acute pancreatitis (AP) globally [1,2,3], and a major driver of the rising global AP incidence due to changing dietary patterns and increasing prevalence of metabolic syndrome [4,5,6,7]. Diagnosed by serum triglyceride levels exceeding 11.3 mmol/L (or 5.65–11.3 mmol/L with chylous blood) [8,9]. HTG-AP is associated with significant clinical challenges. These include a high rate of severe disease (22.2%), frequent recurrence (16–32%), substantial complications, and considerable healthcare costs. Critically, the pathogenesis of HTG-AP remains poorly understood, and the consequent lack of effective targeted therapies contributes to poor outcomes in severe cases, underscoring an urgent unmet need for mechanistic and therapeutic research.

Intestinal barrier dysfunction and oxidative stress are pivotal events in the progression of HTG-AP, initiating a vicious cycle of injury [10,11,12]. The systemic inflammation characteristic of HTG-AP disrupts the gut flora, reducing beneficial bacteria (e.g., *Bifidobacterium*, *Bacteroides*) while promoting the overgrowth of pathogens (e.g., *Enterococcus*, *Escherichia*) [13,14,15,16,17], Concurrently, it directly impairs the intestinal epithelial barrier by downregulating tight junction proteins such as zonula occludens-1 (ZO-1) and Occludin (OCLN) [18] and intestinal oxidative stress response, leading to increased intestinal permeability [19]. This breakdown facilitates bacterial translocation, which, via gut-pancreas axis crosstalk, amplifies pancreatic inflammation and aggravates the disease [20,21,22,23].

Accumulating evidence suggests that endogenous metabolites can modulate intestinal barrier function, regulate gut flora composition, and influence gut-pancreas axis crosstalk, thereby affecting the severity and prognosis of AP [24]. Phenyllactic acid (PLA), a gut microbiota-derived metabolite, has demonstrated anti-inflammatory, antioxidant, and microbiota-regulating properties in various disease models [24,25]. Notably, PLA has been shown to protect intestinal mucosal integrity and promote a balanced microbial community [25]. However, its role in HTG-AP remains completely unexplored.

C57BL/6J mice are a well-established strain for constructing metabolic pancreatitis models, and the poloxamer 407 (P-407) combined with CER-induced HTG-AP model closely recapitulates the pathological features of clinical human HTG-AP [26]. Using this validated mouse model, in this study, we tested the hypothesis that PLA protects against experimental HTG-AP by preserving intestinal epithelial barrier integrity and antioxidant stress response through a peroxisome proliferator-activated receptor gamma (PPARγ)-dependent mechanism. By elucidating this novel pathway, we aim to identify new therapeutic targets for HTG-AP, advance the understanding of gut-pancreas axis regulation, and establish a foundation for developing targeted interventions against this severe disease.

## 2. Materials and Methods

### 2.1. Animal Experiments

Male C57BL/6J mice (specific pathogen-free, 7~8 weeks old, 18~22 g) were purchased from GemPharmatech Co., Ltd. (Nanjing, China), and age-matched littermate controls were used to minimize genetic background variation. All animal experiments were approved by the Animal Management and Welfare Ethics Committee of Jiangnan University (Approval No.: JN.No20240515c0241207 [242], 15 May 2024) and performed in accordance with national animal welfare guidelines, ARRIVE 2.0 guidelines, and AVMA Euthanasia Guidelines. Only male C57BL/6J mice were included to eliminate estrogen-induced biological interference and ensure the stability and repeatability of model construction [9].

After a 1-week acclimatization and health observation in a quarantine facility, mice were housed in a SPF barrier facility (Wuxi School of Medicine, Jiangnan University) under controlled conditions: 20~26 °C, 40~70% humidity, 20~50 Pa static pressure, 10~20 air exchanges/h, 0.1~0.2 m/s air velocity, and 12 h light/dark cycle. Environmental enrichment (nesting material, cardboard tunnels) was provided, with ad libitum access to standard rodent chow and sterile drinking water. Sample size was set at 6~8 mice per group to account for potential modeling-related mortality, and the HTG-AP modeling protocol was performed with reference to previously reported literature [26,27]. Inclusion criteria: healthy mice with normal weight gain and no signs of infection during acclimatization. Exclusion criteria: unexpected death, severe adverse events, or serum triglyceride (TG) < 10 mmol/L post P-407 induction. Mice were randomly assigned to experimental groups using a random number table before modeling, and blinding was applied to investigators performing interventions, outcome assessments and data analysis (unblinded only after complete data collection). 2~3% isoflurane inhalation anesthesia was used for all invasive procedures. Mice were monitored daily for clinical signs of distress (e.g., lethargy, hunched posture, >15% weight loss); the humane endpoint was defined as severe, unresponsive distress, and mice meeting this criterion were humanely euthanized via CO_2_ inhalation (flow rate: 30~70% chamber volume/minute) followed by cervical dislocation to confirm death, in accordance with AVMA guidelines.

The combination of P-407 and caerulein is a well-established and widely used model for HTG-AP, capable of inducing sustained hypertriglyceridemia alongside acute pancreatic injury [26,27]. A detailed experimental timeline and strict validation criteria were adopted to ensure the reproducibility of the study. All animal experiments were conducted in accordance with a randomized and single-blinded design: animals were randomly divided into different groups using a random number table, and the researchers responsible for sample collection, index detection, and data analysis were kept blinded to the group assignments to eliminate subjective bias. The specific procedures are as follows: (1) HTG induction by intraperitoneal (i.p.) injection of P-407 (Cat#P4344419, Aladdin, 0.5 g/kg, Shanghai, China) every other day for 28 d (validated by serum TG ≥ 10 mmol/L on d 28); (2) AP induction by hourly i.p. injection of caerulein (CER, Cat#C305198, Aladdin, 50 μg/kg, Shanghai, China) for 10 consecutive times on d 29 (validated by elevated serum lipase and typical pancreatic pathological changes). For PLA intervention, the doses of PLA were selected based on previously published studies with appropriate adjustments [25,28]. PLA (dissolved in normal saline) was given by intragastric (i.g.) gavage daily throughout HTG modeling at 17.5, 35, and 70 mg/kg for dose-effect screening. The control group received an equal volume of normal saline via the corresponding route. For the AAV intervention group: Colon-specific PPARγ knockdown was established using HBAAV2/9-villin(15)-mir30-m-Pparg-ZsGreen (serotype AAV9, titer: 1.9 × 10^12^ vg/mL), with HBAAV2/9-villin(15)-ZsGreen (serotype AAV9, titer: 2.2 × 10^12^ vg/mL) serving as the negative control. Both vectors were by Hanheng Biotechnology Co., Ltd., (Shanghai, China). The targeting sequence was designed based on the miR30 scaffold and verified by Sanger sequencing to ensure sequence accuracy. A single dose of AAV vector was administered via colonic enema 21 days prior to HTG model initiation (i.e., on Day 1 of the entire experimental timeline), with a volume of 200 μL per animal. Following a 21-day incubation period to allow for stable viral transduction and transgene expression, HTG modeling and subsequent AP induction were performed from Day 22 to Day 40 of the experiment. At the end of the study, the infection efficiency was evaluated by detecting the fluorescence intensity of the reporter gene (ZsGreen) in colonic tissues using fluorescence microscopy. The knockdown efficiency of PPARγ was further confirmed by Western blot analysis, with a reduction in colonic PPARγ protein levels serving as the validation criterion. The Mir30-based shRNA sequence targeting mouse Pparg was amplified by overlap PCR using the following primers:
AAV-Mir30-m-Pparg-F1tccgccttgccctaagaattcgatatcAAGGTATATTGCTGTTGACAGTGAGCGAAV-Mir30-m-Pparg-R1TGTGGCTTCACTAATACAAATGCTTTGCCAGGGCCGCTCACTGTCAACAGCAATAAV-Mir30-m-Pparg-F2TTTGTATTAGTGAAGCCACAGATGTAATACAAATGCTTTGCCAGGGCTGCCTACAAV-Mir30-m-Pparg-R2atccagaggttgattatcgataagcttCGAGGCAGTAGGCAGCCCTGGCAAAGC

For the rosiglitazone (RSG, Cat#122320-73-4, TargetMol, 10 mg/kg, Shanghai, China) positive control group, RSG was given by daily i.g. gavage in the final week of HTG modeling, with an additional i.p. injection 3 h before HTG-AP induction [29].

Three hours after the final intervention, all mice were humanely euthanized via CO_2_ inhalation (flow rate: 30~70% chamber volume/minute) followed by cervical dislocation to confirm death. Peripheral blood, pancreatic/colonic tissues, and colonic contents were collected and aliquoted. Pancreatic/colonic tissues were split into two parts: one fixed in 4% paraformaldehyde for 24 h for paraffin embedding (histopathological analysis), and the other snap-frozen in liquid nitrogen and stored at −80 °C (transcriptomic and molecular analyses). Serum was separated from peripheral blood by centrifugation (3000× *g*, 15 min, 4 °C), and all samples were stored at −80 °C until analysis.

The modeling procedures and sample collection protocols were consistent across all groups to ensure comparability of results. Intervention timings, sample collection time points pancreatic and colonic tissues were collected on day 29 after the initiation of HTG modeling, and detection methods were consistent across all groups to ensure the comparability of experimental results.

### 2.2. Detection of Serum Biochemical Indicators

Mouse blood samples were collected via the orbital venous plexus and allowed to stand at room temperature for 30 min. Samples were then centrifuged at 3000 r/minute for 15 min to separate the serum. The obtained serum was aliquoted into cryovials, labeled with group information, collection time, and other relevant details, and stored at −80 °C in an ultra-low temperature refrigerator for subsequent use. Serum levels of lipase (LIP), TG, and total cholesterol (TC) were detected using an automatic biochemical analyzer at the State Key Laboratory of Food Science and Resources, Jiangnan University.

### 2.3. Collection of Clinical Serum Samples

This study retrospectively collected anonymous residual serum specimens from routine clinical examinations, without additional invasive operations, clinical interventions, or increased risks to patients. Specimens were obtained from patients diagnosed with HTG-AP admitted to the Department of Hepatobiliary Surgery, the Affiliated Hospital of Jiangnan University, between February 2024 and May 2026. Age- and gender-matched healthy individuals who received regular physical examinations during the same period were enrolled as the control group.

All sample collection protocols were approved by the Medical Ethics Committee of the Affiliated Hospital of Jiangnan University (Approval No.: LS2026042, 9 April 2026). Formal exemption from written informed consent was granted by the ethics committee, in full compliance with the Declaration of Helsinki and relevant ethical regulations for human residual specimen research.

The diagnostic criteria for HTG-AP referred to the 2021 Chinese Guidelines for the Diagnosis and Treatment of Acute Pancreatitis (2021 Edition) [30], including serum triglyceride (TG) ≥ 11.3 mmol/L, or TG ranging from 5.65 to 11.3 mmol/L accompanied by chylous blood, combined with acute abdominal pain, serum lipase elevation exceeding three times the upper limit of the normal range, and pancreatic inflammatory changes confirmed by imaging. Fasting residual venous blood samples from HTG-AP patients were collected within 24 h of admission before initiation of lipid-lowering, anti-inflammatory or other therapeutic interventions. Healthy control subjects had no history of pancreatic diseases or chronic metabolic disorders (e.g., hyperlipidemia, diabetes mellitus), with serum TG < 1.7 mmol/L and normal liver, renal, and routine blood test indicators. All serum samples used in the present study were leftover specimens after conventional clinical testing and were anonymized to protect patient privacy. All samples were collected under unified inclusion criteria and ethical approval, and within the defined pre-treatment window prior to lipid-lowering intervention.

### 2.4. Liquid Chromatography-Tandem Mass Spectrometry (LC-MS/MS) Detection

PLA and the internal standard (DL-3-phenyllactic acid-d_3_) were dissolved in acetonitrile to prepare a series of standard solutions. The internal standard concentration was fixed at 10 ng/mL, while PLA was prepared in a concentration gradient ranging from 10^−1^ to 10^−6^ mg/mL. Solutions were filtered through a 0.22 μm membrane prior to detection, and a standard curve was generated based on the peak area-concentration relationship using six calibration points.

Intestinal content samples: 20 mg of each sample were accurately weighed and mixed with 0.5 mL of a water/acetonitrile mixture (3:2, *v*/*v*) to prepare a 40 mg/mL suspension. 2~3 steel beads were added, and samples were homogenized using a bead mill. Homogenates were centrifuged at 12,000 rpm for 15 min at 4 °C, and the supernatant was collected. Subsequently, 180 μL of the supernatant was mixed with 20 μL of 100 ng/mL internal standard solution (final concentration 10 ng/mL), centrifuged at 12,000 rpm for 5 min at 4 °C, and filtered prior to LC-MS/MS analysis. Serum samples: 20 μL of serum were mixed with 160 μL of acetonitrile and 20 μL of 100 ng/mL internal standard solution (final concentration 10 ng/mL). The mixture was vortexed thoroughly, centrifuged at 12,000 rpm for 15 min at 4 °C, and the supernatant was filtered through a 0.22 μm membrane before detection.

All analyses were performed at the Analysis and Testing Center of Jiangnan University using a triple quadrupole linear ion trap LC-MS/MS system operated in multiple reaction monitoring (MRM) mode. Chromatographic separation was carried out on an Agilent HSS-T3 column (1.8 μm, 2.1 × 100 mm, Santa Clara, CA, USA) with a column temperature of 40 °C, an injection volume of 2 μL, and a flow rate of 0.3 mL/minute. The mobile phase consisted of 0.1% formic acid in water (phase A) and acetonitrile (phase B), with the following gradient program: 0~1 min, 95% A; 1~4 min, linearly decreased to 5% A; 4~6 min, maintained at 5% A; 6~6.1 min, linear increase back to 95% A; 6.1~9 min, maintained at 95% A. Mass spectrometry data were collected in negative ion mode. The processing methods were performed in accordance with the study reported by Shelton et al. [28,31]. Key reagents are listed in Table S1.

### 2.5. Determination of Pancreatic Tissue Edema

Following anesthesia with isoflurane inhalation, pancreatic tissue was collected by laparotomy under sterile conditions. Residual blood and abdominal fluid on the tissue surface were gently blotted with pre-cooled sterile absorbent filter paper, and the wet weight was measured using an electronic analytical balance. Tissue were then transferred to a 60 °C electric constant-temperature blast drying oven for 72 h. After cooling to room temperature in a desiccator, and the dry weight was recorded. The wet-to-dry weight ratio was calculated to quantitatively assess the degree of pancreatic edema.

### 2.6. Histopathological Staining

Following anesthesia and euthanasia with isoflurane, pancreatic and colonic tissues were fixed in 4% paraformaldehyde for 24~48 h, rinsed with running water for 2 h, dehydrated with gradient ethanol, cleared with xylene, and embedded in paraffin. 4 μm paraffin sections were prepared using a Leica microtome (Wetzlar, Germany). After deparaffinization and rehydration with xylene and gradient ethanol, periodic acid-Schiff staining (PAS) and hematoxylin-eosin staining (HE) were performed in accordance with the respective kit instructions. Tissue morphology was observed using a digital slice scanner (Pannoramic, 3DHISTCH, Budapest, Hungary). Histopathological evaluation was conducted based on scoring criteria from previous studies [32,33]. Pancreatic tissue scoring standard: 0 points (normal, no damage), 2 points (edema, intact structure), 4 points (slightly widened lobular space, accompanied by lipid vacuoles), 6 points (inflammatory exudation and inflammatory cell infiltration), 8 points (acinar cell necrosis, severe hemorrhage), 10 points (tissue structure destruction, disordered cell arrangement). Colon tissue scoring standard: 0 points (normal, no damage), 2 points (gland tip rupture, mild separation), 4 points (gland rupture, submucosal edema, hemorrhage), 6 points (muscular layer hyperplasia and thickening, gland shortening), 8 points (submucosal separation, weakened crypt hyperplasia), 10 points (transmural injury, severe gland shortening). Histopathological evaluation was performed by at least two independent experienced pathology researchers who were fully blinded to the experimental grouping and treatment conditions to eliminate subjective assessment bias.

### 2.7. Immunofluorescence Staining

Paraffin sections were deparaffinized and rehydrated sequentially with xylene and graded ethanol. Antigen retrieval was performed by immersing the section in 1 × Tris-EDTA buffer (pH = 9.0) and heating under high-temperature, high-pressure steam (95~100 °C, 30 min). Sections were then treated with 0.5% permeabilization solution, blocked with 5% bovine serum albumin, and incubated with primary antibodies overnight at 4 °C. Primary antibodies used: F4/80 (Cat#28463-1-AP, 1:200) was purchased from Proteintech Biotechnology (Wuhan, China). On the following day, sections were equilibrated to room temperature for 30 min, counterstained with 4′,6-diamidino-2-phenylindole (DAPI) for 10 min, and mounted with anti-fade immunofluorescence mounting medium. Images were acquired using a Zeiss Axio Imager2 fluorescence microscope (Oberkochen, Germany).

### 2.8. Western Blot Analysis (WB)

Colon tissue (30 mg) were thawed and lysed with RIPA buffer containing phosphatase and protease inhibitors at a ratio of 1 mg tissue:10 μL buffer. Tissues were homogenized using magnetic beads (60 Hz, 30 s/time, 4~5 times) and incubated on ice for 30 min. Lysates were centrifuged at 12,000× *g* at 4 °C for 15 min, and the supernatant was collected. Protein concentrations were determined using the bicinchoninic acid (BCA) assay, and samples were normalized accordingly. Protein samples were mixed with loading buffer, heated at 100 °C for 10 min, and stored at −80 °C for later use.

Equal amounts of protein were separated by SDS-polyacrylamide gel electrophoresis and transferred onto nitrocellulose membranes by wet transfer. Membranes were blocked with 5% skimmed milk at room temperature for 2 h, incubated overnight at 4 °C with primary antibodies, and subsequently incubated with HRP-conjugated secondary antibodies at room temperature for 2 h the next day, with TBST washes performed throughout. Chemiluminescence detection was carried out using equal volumes of ECL developer solutions A and B, and the results were recorded. Primary antibodies used: ZO-1 (Cat#21773-1-AP, 1:1000), Zonula occludens-2 (ZO-2, Cat#18900-1-AP, 1:1000) were purchased from Proteintech Biotechnology (Wuhan, China); OCLN (Cat#AP0765, 1:1000) was purchased from Biogot Technology (Nanjing, China); Claudin 1 (CLDN1, Cat#4933T, 1:1000) was purchased from Cell Signaling Technology (Danvers, MA, USA); Peroxisome Proliferator-Activated Receptor Gamma (PPARγ, Cat#S0B0506, 1:1000) was purchased from Starter Biotechnology (Hangzhou, China). β-actin (Cat#ZB15001-HRP-100, 1:5000, Servicebio Technology, Wuhan, China) was used as the internal control. Protein bands intensities were quantitatively analyzed using ImageJ software 10.1.2 version. (NIH, Bethesda, MD, USA).

### 2.9. Enzyme-Linked Immunosorbent Assay (ELISA)

The levels of lipopolysaccharide (LPS) in mouse serum were measured using ELISA kits (Cat#YJ022423B, Solarbio Science & Technology, Beijing, China) according to the manufacturer’s instructions. Frozen pancreatic tissue (20~30 mg) was homogenized on ice in pre-cooled phosphate buffer saline (PBS) containing protease inhibitors at a ratio of 1:9 (tissue:buffer, *w*/*v*). Homogenates were centrifuged at 12,000 rpm at 4 °C for 15 min, and the supernatant was collected. pancreatic MPO levels were then determined using ELISA kits (Cat#SEKM-0118, Enzyme-linked Biotechnology, Shanghai, China) according to the manufacturer’s protocol.

### 2.10. 16S rDNA Gene Sequencing

The 16S rDNA gene sequencing was performed at the State Key Laboratory of Food Science and Resources, Jiangnan University. The V3–V4 hypervariable region of bacterial 16S rDNA gene was amplified using the following primers: 341F (5′-CCTAYGGGRBGCASCAG-3′) and 806R (5′-GGACTACNNGGGTATCTAAT-3′). Subsequent sequencing and flora analysis were performed according to standard procedures.

### 2.11. Fluorescence In Situ Hybridization (FISH)

Pancreatic and colonic tissues were dehydrated, embedded, sectioned, deparaffinized, and rehydrated to obtain suitable sections. Sections were then permeabilized with proteinase K solution and blocked with hybridization solution containing 20% formamide. Specific in situ hybridization fluorescent probe EUB338 (5′-Cy3-GCTGCCTCCCGTAGGAGT-3′) (Servicebio Technology, Wuhan, China) were applied dropwise at concentrations adjusted according to the kit instructions, and sections were incubated at 4 °C. The following day, sections were rinsed with graded saline-sodium citrate buffer, counterstained with FISH-specific DAPI solution for nuclear visualization, rinsed with PBS, and mounted. Images were acquired using a Zeiss Axio Imager2 fluorescence microscope (Oberkochen, Germany).

### 2.12. Quantitative Real-Time Polymerase Chain Reaction (qRT-PCR)

Approximately 20 mg of fresh mouse pancreatic or colonic tissue was used for RNA extraction. Total RNA was isolated using TRIzol reagent (Cat#19201ES60, Yeasen Biotechnology, Wuhan, China), followed by chloroform extraction, isopropanol precipitation, and 75% ethanol washing, and subsequently dissolved in DEPC-treated water. RNA concentration and purity were determined using an ultra-micro ultraviolet spectrophotometer, with OD_260_/OD_280_ ratios ranging from 1.8 to 2.0. RNA concentrations were standardized to 500 ng/μL. Complementary DNA (cDNA) was synthesized by reverse transcription according to the manufacturer’s instructions and stored at −20 °C until use. Specific primers synthesized by Sangon Biotech (Shanghai) were used for qRT-PCR reactions, with three biological replicates for each sample. The relative expression levels of target genes were calculated using the 2^−ΔΔCt^ method. Primer information for RT-qPCR is provided in Table S2.

### 2.13. Frozen Section Preparation and Viral Immunofluorescence Verification

Fresh colonic tissues were fixed in 4% paraformaldehyde at 4 °C for 24 h, followed by dehydration in graded sucrose solutions (10%, 20%, 30%). Complete dehydration was confirmed by tissue sinking, with timely replacement of the sucrose solutions. Tissues were then embedded in optimal cutting temperature compound and frozen at −80 °C. Frozen sections (8 μm) were prepared, baked at 60 °C for 1 h, sealed, and stored at −80 °C until use.

Prior to staining, sections were equilibrated to room temperature and air-dried, washed with PBS, and permeabilized with 0.5% Triton X-100 at room temperature for 20~30 min. After washing again with PBS, sections were mounted with anti-fade mounting medium containing DAPI. Images were acquired using a Zeiss upright fluorescence microscope to verify the AAV infection efficiency and to verify PPARγ gene silencing in colonic tissues.

### 2.14. Immunohistochemistry

Following antigen retrieval (per the standard protocol for paraffin-embedded tissue immunofluorescence), sections were allowed to cool to room temperature, rinsed in distilled water, and washed twice with PBS for 3 min each. Immunohistochemical staining was performed using the Rabbit/Mouse Universal Streptavidin-HRP Kit (DAB) (Cat#CW2069S, CWBIO, Taizhou, China) according to the manufacturer’s instructions. Briefly, endogenous peroxidase activity was blocked by incubation with Solution A at room temperature for 10 min, followed by PBS washing. Non-specific binding was blocked by incubation with Solution B (normal goat serum) at room temperature for 10 min. Sections were then incubated with the primary antibody against PPARγ (Cat#S0B0506, 1:200 dilution, Starter Biotechnology, Hangzhou, China) at the optimized condition, washed thoroughly with PBS, and incubated with biotin-conjugated goat anti-rabbit/anti-mouse secondary antibody (Solution C) at room temperature for 10 min, followed by PBS washing. After incubation with HRP-conjugated streptavidin (Solution D) at room temperature for 10 min and PBS rinsing, immunoreactivity was visualized with 3,3′-diaminobenzidine (DAB) working solution (DAB-A:DAB-B = 19:1). The chromogenic reaction was monitored microscopically for 1~5 min and stopped by washing with tap water. Sections were counterstained with hematoxylin, dehydrated, cleared in xylene, and mounted for imaging.

### 2.15. RNA Sequencing (RNA-Seq)

Colon tissue specimens were prepared for total RNA extraction prior to transcriptomic profiling. Library construction and high-throughput sequencing were performed by Shanghai HonsunBio Technology Co., Ltd. (Shanghai, China) using the Illumina NovaSeq 6000 platform (San Diego, CA, USA). After strict quality control and data filtering, the obtained high-quality clean reads were aligned to the Mus musculus reference genome, with annotated gene sequence files retrieved from the NCBI database. Differentially expressed genes (DEGs) were identified using standardized statistical thresholds. The resulting gene expression profiles were subjected to hierarchical clustering, heatmap visualization, and Kyoto Encyclopedia of Genes and Genomes (KEGG) pathway enrichment analysis. For KEGG pathway enrichment analysis, pathways were considered significantly enriched when the adjusted *p*-value < 0.05 and fold change > 2 or <0.05. The data reported in this paper have been deposited in the OMIX, China National Center for Bioinformation/Beijing Institute of Genomics, Chinese Academy of Sciences (https://ngdc.cncb.ac.cn/omix/release/OMIX016081, accessed on 6 April 2026).

### 2.16. Superoxide Dismutase (SOD) Activity Detection

SOD activity was measured using the SOD assay kit (Cat#S0101S, Beyotime Biotechnology, Shanghai, China) per the manufacturer’s protocols. Briefly, colon tissues were collected after perfusion with 0.9% NaCl (containing 0.16 mg/mL heparin sodium) for blood removal. Tissues (10 mg) were homogenized in 100 μL SOD sample preparation solution at 4 °C (or ice bath) using glass or electric homogenizers. Homogenates were centrifuged at 12,000× *g*, 4 °C for 3~5 min, and supernatants were used as test samples. Test samples and reagents were added sequentially; after adding the reaction initiation solution, the mixture was thoroughly mixed. SOD activity was calculated as: SOD activity unit in test sample = [inhibition rate/(1 − inhibition rate)] units.

### 2.17. Reactive Oxygen Species (ROS) Detection

ROS levels in colon tissue frozen sections were detected using the ROS assay kit (DCFH-DA method, Cat#S0033, Beyotime Biotechnology, Shanghai, China) following the manufacturer’s instructions. Briefly, sections were fixed with 4% paraformaldehyde for 10 min at room temperature, then washed 3 times with PBS for 5 min each. The DCFH-DA probe (diluted to the recommended concentration with PBS) was added dropwise to cover the sections, followed by incubation in a dark environment at 37 °C for 20~30 min. After incubation, sections were washed 3 times with PBS to remove excess probe. Nuclei were counterstained with DAPI for 5 min, then washed again with PBS. Finally, sections were mounted with anti-fluorescence quenching mounting medium and observed under a fluorescence microscope. ROS levels were quantified by measuring the mean fluorescence intensity of positive areas using image analysis software.

### 2.18. Molecular Docking

The crystal structure of human PPARγ ligand-binding domain (LBD) was retrieved from the Protein Data Bank (PDB ID: 2POB, resolution: 2.30 Å, https://www.rcsb.org/, accessed on 29 April 2026). Chain A of the PPARγ crystal structure was selected for subsequent molecular docking. Water molecules and redundant heteroatoms were removed from the raw protein structure, and polar hydrogen atoms as well as Gasteiger partial charges were added for structural optimization using PyMOL software (v2.2.0). The 2D structure of PLA was acquired from the PubChem database (PubChem CID: 3848), and further converted into a 3D structural format with Open Babel (2.4.1). Molecular docking between PLA and PPARγ was performed using AutoDock Vina (v1.2.5). The grid box was centered on the ligand-binding pocket of PPARγ with dimensions set to 40 × 40 × 40 Å. The exhaustiveness was set to 32, and a total of 100 independent docking runs were conducted. The binding affinity (kcal/mol) was applied to evaluate the binding capacity between PLA and PPARγ. The optimal conformation with the lowest docking energy was selected for subsequent interaction analysis and visualized by PyMOL. Non-covalent molecular interactions between PLA and PPARγ were further characterized using the Protein-Ligand Interaction Profiler online tool (https://plip-tool.biotec.tu-dresden.de/plip-web/plip/index, accessed on 29 April 2026).

### 2.19. Statistical Analysis

All analyses were performed using GraphPad Prism 10.0, and a unified statistical framework has been consistently applied in the Methods, main text, and all figure legends. Specifically, data distribution was first assessed using the Shapiro–Wilk normality test, and homogeneity of variance was evaluated where appropriate. For normally distributed data, comparisons between two groups were performed using unpaired Student’s *t*-test, while multiple group comparisons were conducted using one-way ANOVA followed by Tukey’s post hoc test. For non-normally distributed data, non-parametric tests (e.g., Mann–Whitney U test or Kruskal–Wallis test with Dunn’s post hoc correction) were applied.

All quantitative data are now consistently presented as mean ± SEM, and this format has been uniformly adopted across all figures and legends. We selected SEM to reflect the precision of the estimated mean across biological replicates, given the relatively small sample size (typically *n* = 6 per group) Statistical significance was defined as * *p* < 0.05, ** *p* < 0.01, and *** *p* < 0.001.

For multiple group comparisons, Bonferroni correction was applied to control type I error in routine phenotypic analyses. For high-throughput datasets, including RNA-seq and microbiome analyses, false discovery rate (FDR) was controlled using the Benjamini–Hochberg method. RNA-seq differential expression analysis was performed using DESeq2 (v1.46.0) with FDR-adjusted *p*-values.

### 2.20. Bioinformatics Analysis

Bioinformatics analysis was performed to process and interpret the sequencing data generated in this study. KEGG pathway enrichment analysis of differentially expressed genes from RNA-seq transcriptome data was conducted using the OE Biotech Cloud Platform (OE Biotech Co., Ltd., Shanghai, China). For 16S rRNA gene sequencing data, taxonomic annotation, microbial community composition analysis, and visualization were completed relying on the analytical services of LC-Bio Technology (Lianchuan Bio Technology Co., Ltd., Hangzhou, China) and the Biocloud Platform (Shenzhen Micromeng Technology Group Co., Ltd., Shenzhen, China). All scientific figures involved in this study, including microbial community diagrams and other visualization graphs, as well as the graphical abstract, were generated and edited using the free academic version of Figdraw cloud drawing platform (https://www.figdraw.com, accessed on 12 December 2025), with the official copyright certification ID: OARWOf105f. All bioinformatics analyses were performed in accordance with standard academic protocols for non-commercial research purposes.

## 3. Results

### 3.1. PLA Supplementation Alleviates HTG-AP Severity

**Figure 1:**
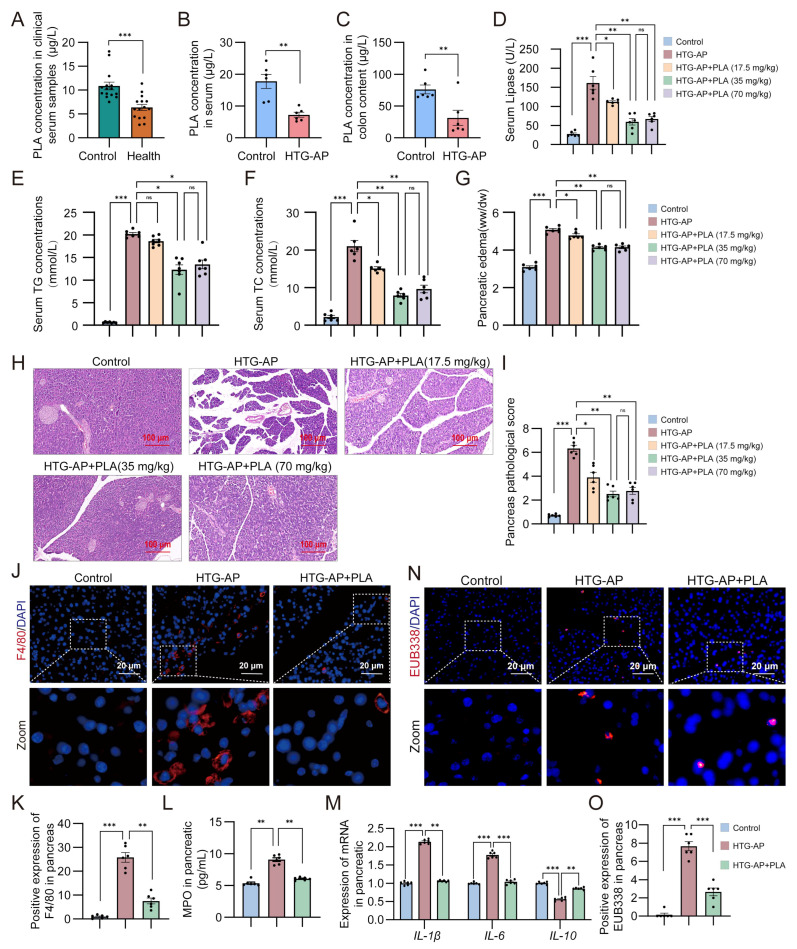
PLA supplementation alleviates HTG-AP severity. (**A**) PLA levels in clinical serum samples, n = 15 per group. (**B**) Serum PLA levels in mice, n = 6 per group. (**C**) PLA levels in mouse colonic contents, n = 6 per group. (**D**) Serum LIP levels, n = 6 per group. (**E**) Serum TG levels, n = 6 per group. (**F**) Serum TC levels, n = 6 per group. (**G**) Pancreatic wet-to-dry weight ratio, n = 6 per group. (**H**,**I**) Representative pancreatic HE staining and histological score statistics. Scale bar: 100 μm, n = 6 per group. (**J**,**K**) Immunofluorescence staining and quantification of F4/80 (a macrophage marker) in pancreas. Scale bar: 20 μm, n = 6 per group. Blue: DAPI, red: F4/80. (**L**) Pancreatic MPO levels determined by ELISA, n = 6 per group. (**M**) Pancreatic mRNA expression levels of *IL-1β*, *IL-6* and *IL-10*, n = 6 per group. (**N**,**O**) Representative EUB338/DAPI staining and quantification in pancreatic sections. Scale bar: 20 μm, n = 6 per group. Blue: DAPI, red: EUB338. (ns, *p* > 0.05; * *p* < 0.05; ** *p* < 0.01; *** *p* < 0.001).

To investigate the correlation between endogenous PLA abundance and HTG-AP pathogenesis, we quantified its levels using LC-MS/MS. Serum PLA was significantly lower in HTG-AP patients compared to healthy controls (Figure 1A and Figure S1). This finding was recapitulated in a murine model of HTG-AP, where both serum and fecal PLA levels were markedly decreased relative to control mice (Figure 1B,C). Collectively, these data suggest that reduced endogenous PLA represents a unique metabolic perturbation tightly correlated with HTG-AP development The therapeutic efficacy of PLA supplementation in HTG-AP mice was evaluated. HTG-AP model mice exhibited profound dyslipidemia, characterized by elevated serum LIP, TG, and TC. PLA supplementation dose-dependently ameliorated these lipid disturbances (Figure 1D–F). Specifically, treatment with 35 mg/kg PLA significantly reduced serum LIP and TG levels, while both 35 mg/kg and 70 mg/kg PLA significantly lowered TC levels (Figure 1D–F). PLA treatment markedly attenuated pancreatic edema and tissue injury (Figure 1G–I). Consistent with this protective effect, PLA supplementation reversed the HTG-AP-associated increase in pancreatic macrophage and neutrophil infiltration. This was accompanied by a significant reduction in the expression of pro-inflammatory cytokines *IL-1β* and *IL-6* and a restoration of the anti-inflammatory cytokine *IL-10* (Figure 1J–M). FISH analysis revealed PLA supplementation attenuated bacterial penetration into pancreatic tissues in HTG-AP mice (Figure 1N,O). Collectively, these results demonstrate that endogenously derived metabolite PLA levels are reduced during HTG-AP progression and that exogenous PLA supplementation exerts a protective effect in HTG-AP mice.

### 3.2. PLA Alleviates Colonic Epithelial Injury in HTG-AP Mice

**Figure 2:**
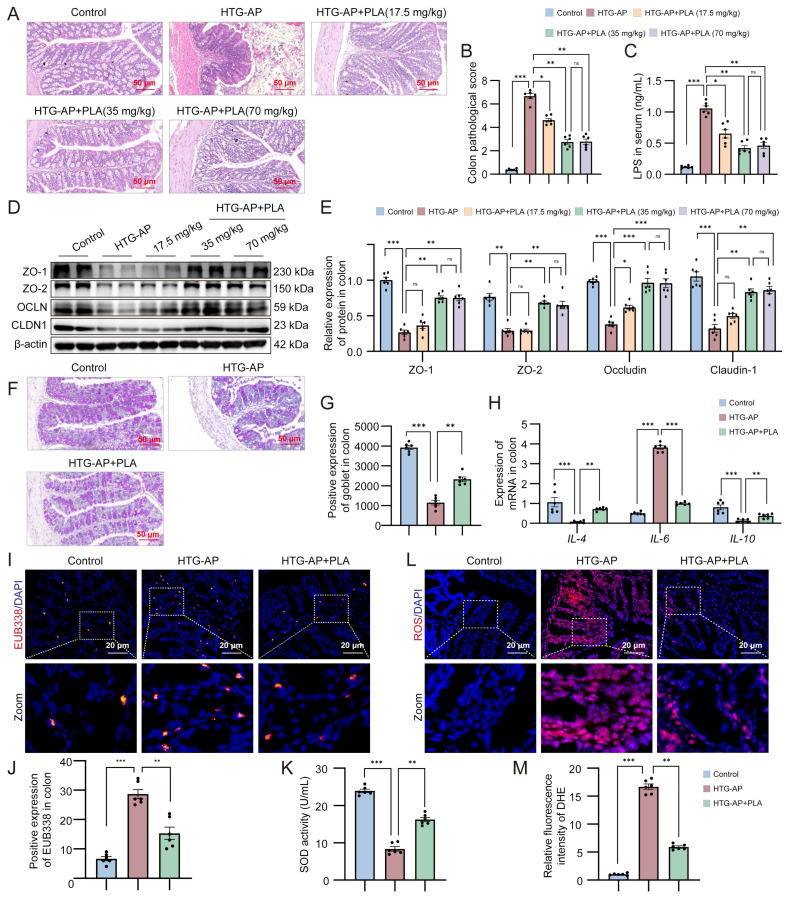
PLA alleviates colonic epithelial injury in HTG-AP mice. (**A**,**B**) Representative colonic HE staining images and histological scores. Scale bar: 50 μm, n = 6 per group. (**C**) Serum LPS levels, n = 6 per group. (**D**,**E**) WB analysis of tight junction proteins ZO-1, ZO-2, OCLN, and CLDN1 in colon, with quantification of their relative expression levels; β-actin served as the internal reference, n = 6 per group. (**F**,**G**) Representative PAS staining of colonic goblet cells and statistical analysis. Scale bar: 50 μm, n = 6 per group. (**H**) Colonic mRNA expression levels of *IL-4*, *IL-6* and *IL-10*, n = 6 per group. (**I**,**J**) Representative EUB338/DAPI staining images and quantification in colonic sections. Scale bar: 20 μm, n = 6 per group. Blue: DAPI, red: EUB338. (**K**) Serum SOD levels, n = 6 per group. (**L**,**M**) Representative ROS/DAPI staining images and quantification in colonic sections. Scale bar: 20 μm, n = 6 per group. Blue: DAPI, red: ROS. (ns, *p* > 0.05; * *p* < 0.05; ** *p* < 0.01; *** *p* < 0.001).

Given the pronounced intestinal structural and functional impairments observed in HTG-AP mice and their established role in gut-pancreas axis crosstalk [20]. The colonic histopathology and barrier function was evaluated. H&E staining revealed that HTG-AP induction caused severe colonic injury, characterized by marked glandular atrophy, crypt destruction, goblet cell loss and focal epithelial injury, which was improved by PLA supplementation a dose-dependent manner (Figure 2A). Consistently, Western blot analysis showed that the expression of tight junction proteins (ZO-1, ZO-2, OCLN, and CLDN1) was significantly decreased in HTG-AP mice, accompanied by elevated serum LPS levels. PLA treatment restored the expression of these tight junction proteins and reduced circulating LPS levels (Figure 2C–E). PAS staining revealed a significant reduction in goblet cell numbers in HTG-AP mice, which was markedly reversed by PLA supplementation (Figure 2F,G). Moreover, PLA treatment significantly attenuated the HTG-AP-associated elevation of *IL-6* and restored the levels of *IL-4* and *IL-10* in colonic tissues (Figure 2H). FISH analysis revealed increased bacterial penetration into colonic tissues in HTG-AP mice, which was markedly reduced following PLA supplementation (Figure 2I,J). Furthermore, intestinal oxidative stress directly compromises intestinal barrier integrity [34]. HTG-AP elicits intestinal oxidative stress injury and diminishes systemic antioxidant capacity, whereas PLA mitigates this redox imbalance by suppressing colonic ROS generation and augmenting SOD activity (Figure 2K–M). Collectively, these results demonstrate that PLA supplementation protects against colonic epithelial injury in HTG-AP by restoring tight junction integrity.

### 3.3. PLA Alleviates Gut Dysbiosis in HTG-AP Mice

**Figure 3:**
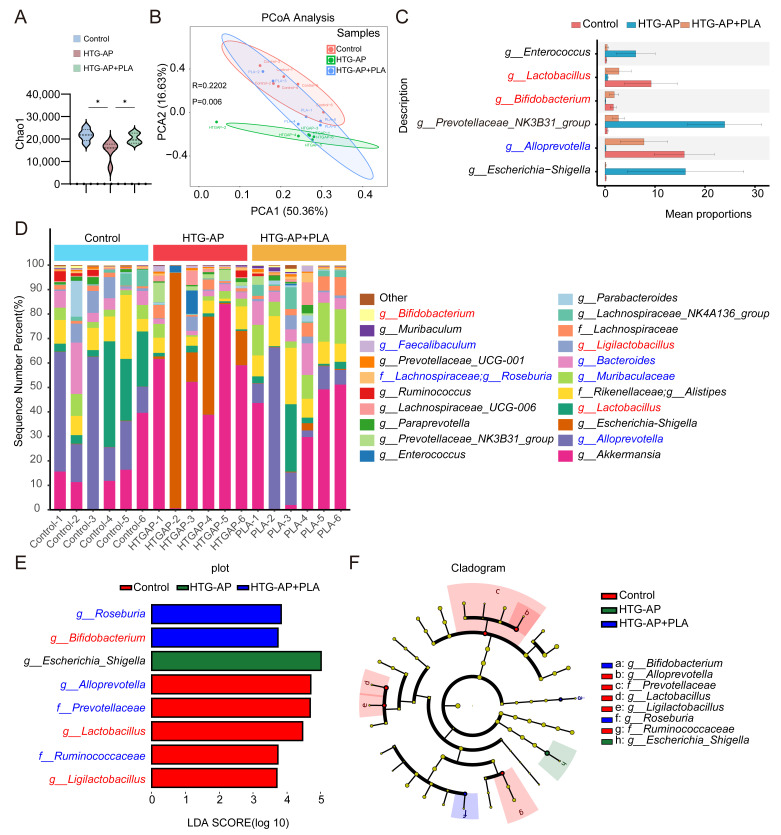
PLA alleviates gut dysbiosis in HTG-AP mice. (**A**) Alpha diversity analysis of the intestinal microbiota, as measured by the Chao1 index. Asterisks denote significant differences between groups, n = 6 per group. (**B**) PCoA of intestinal microbiota profiles, n = 6 per group (R^2^ = 0.2202, *p* = 0.006). (**C**) Mean relative abundance of key bacterial genera in each group, n = 6 per group. (**D**) Stacked bar chart showing the relative abundance of bacterial taxa at the family and genus levels in individual samples, n = 6 per group. (**E**) Linear discriminant analysis effect size (LEfSe), using an LDA score threshold of >3, n = 6 per group. (**F**) Cladogram visualizing the phylogenetic distribution of differentially abundant taxa identified by LEfSe, n = 6 per group. (* *p* < 0.05). (**C**–**E**). Red: taxa previously implicated in PLA biosynthesis; Blue: taxa previously implicated in short-chain fatty acid production; Black: other bacterial taxa.

Given that gut dysbiosis also play a critical role in HTG-AP pathogenesis, we next investigated the regulatory effect of PLA on the gut microbial community. α-diversity analysis revealed a significant reduction in species richness (Chao1 index) in HTG-AP mice compared to controls, which was restored by PLA supplementation (Figure 3A). Principal coordinate analysis (PCoA) based on Bray–Curtis dissimilarity showed microbial community clustering among the three groups (R^2^ = 0.2022, *p* = 0.006), with the PLA-treated group clustering more closely with controls (Figure 3B). Analysis of key genera (Figure 3C,D) showed that the HTG-AP condition was associated with decreased relative abundance of SCFA-producing taxa (e.g., *Alloprevotella*, *Bacteroides*) and several *Lactobacillus* and *Bifidobacterium* species, alongside the enrichment of the potential pathogenic genus *Escherichia-Shigella*. HTG-AP mice presented reduced relative abundances of reported beneficial genera, including SCFA-producing *Alloprevotella* and *Bacteroides* [35], as well as *Lactobacillus* and *Bifidobacterium*—taxa previously implicated in PLA biosynthesis [28,36]. Meanwhile, the opportunistic pathogenic genus. *Escherichia-Shigella* was markedly enriched in the HTG-AP group. Notably, PLA supplementation effectively rescued these microbial perturbations. Effect Size (LEfSe) analysis and taxonomic cladograms (Figure 3E,F) further verified the differential microbial signatures among groups. Taxa such as *Bifidobacterium* (reported to be associated with PLA synthesis) were identified as characteristic biomarkers in the PLA-treated group. SCFA-producing microbes, including *Alloprevotella* and members of the *Ruminococcaceae* family, were enriched in control mice, whereas the pathobiont *Escherichia-Shigella* served as a key signature genus in the HTG-AP model. Together, these findings demonstrate that PLA supplementation concurrently ameliorates gut dysbiosis in HTG-AP mice.

### 3.4. Colonic Epithelial PLA Alleviates HTG-AP Injury Through a PPARγ-Dependent Mechanism

**Figure 4:**
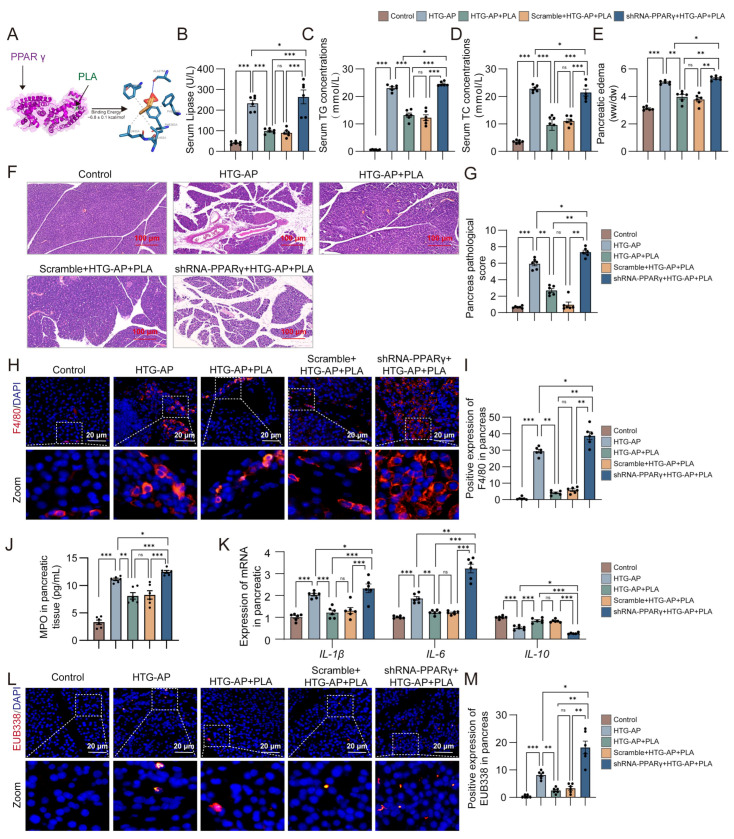
Colonic Epithelial PPARγ is required for PLA-mediated protection in HTG-AP. (**A**) Molecular docking analysis of PLA binding to PPARγ. (**B**) Serum LIP levels, n = 6 per group. (**C**) Serum TG levels, n = 6 per group. (**D**) Serum TC levels, n = 6 per group. (**E**) Pancreatic wet-to-dry weight ratio, n = 6 per group. (**F**,**G**) Representative pancreatic HE staining and histological score statistics. Scale bar: 100 μm, n = 6 per group. (**H**,**I**) Immunofluorescence staining and quantification of F4/80 (a macrophage marker) in pancreas. Scale bar: 20 μm, n = 6 per group. Blue: DAPI, red: F4/80. (**J**) Pancreatic MPO levels determined by ELISA, n = 6 per group. (**K**) Pancreatic mRNA expression levels of *IL-1β*, *IL-6* and *IL-10*, n = 6 per group. (**L**,**M**) Representative EUB338/DAPI staining and quantification in pancreatic sections. Scale bar: 20 μm, n = 6 per group. Blue: DAPI, red: EUB338. (ns, *p* > 0.05; * *p* < 0.05; ** *p* < 0.01; *** *p* < 0.001).

To identify the molecular target of PLA, we first performed KEGG enrichment analysis on downregulated genes from RNA-seq data of colonic tissues from HTG-AP mice. This revealed PPAR signaling as one of the top pathways affected in HTG-AP (Figure S2A). Immunohistochemical staining confirmed a marked reduction in PPARγ expression in the colonic epithelium of HTG-AP mice (Figure S2B). Molecular docking showed that PLA may act as a ligand of PPARγ to modulate its activity (Figure 4A). To determine whether colonic epithelial PPARγ mediates the protective effects of PLA, we selectively silenced its expression using an AAV-delivered shRNA (Figure S2D). Efficient epithelial targeting and PPARγ knockdown were confirmed by fluorescence imaging and Western blotting (Figure S2E–H).

PLA supplementation significantly reduced serum markers of pancreatic injury and dyslipidemia (TG, TC) in HTG-AP mice, which were almost completely abolished upon PPARγ silencing, but not Scramble control (Figure 4B–D). Consistently, colonic epithelial PPARγ knockdown markedly attenuated PLA-mediated improvements in pancreatic histopathology, edema, and inflammatory cell infiltration (Figure 4E–K).

**Figure 5:**
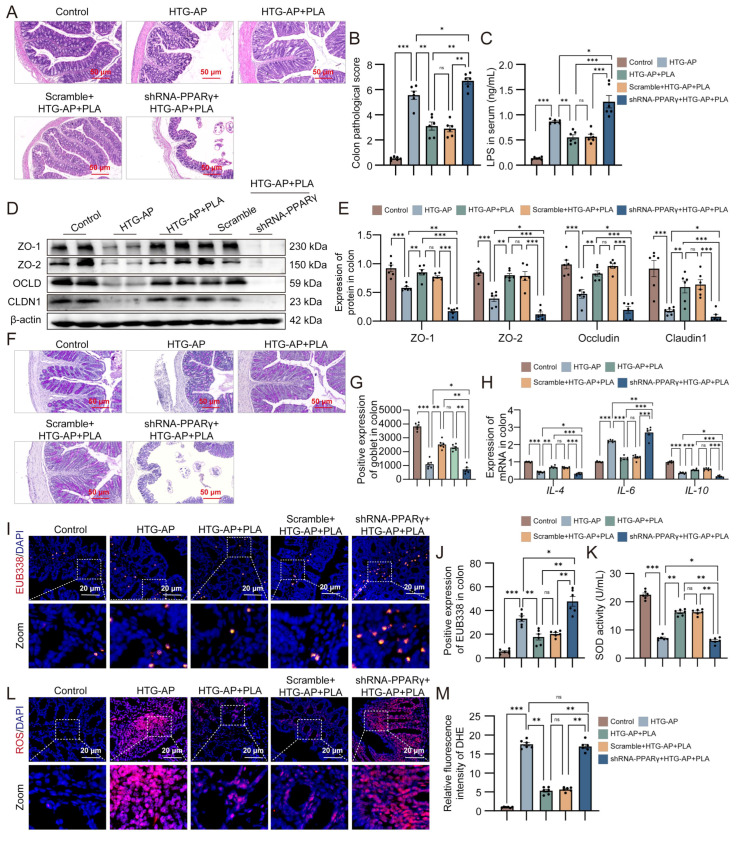
Colonic epithelial PPARγ is indispensable for the protective effect of PLA against colonic epithelial injury in HTG-AP. (**A**,**B**) Representative colonic HE staining images and histological scores. Scale bar: 50 μm, n = 6 per group. (**C**) Serum LPS levels, n = 6 per group. (**D**,**E**) WB analysis of tight junction proteins ZO-1, ZO-2, OCLN, and CLDN1 in colon, with quantification of their relative expression levels; β-actin served as the internal reference, n = 6 per group. (**F**,**G**) Representative PAS staining of colonic goblet cells and statistical analysis. Scale bar: 50 μm, n = 6 per group. (**H**) Colonic mRNA expression levels of *IL-4*, *IL-6* and *IL-10*, n = 6 per group. (**I**,**J**) Representative EUB338/DAPI staining images and quantification in colonic sections. Scale bar: 20 μm, n = 6 per group. Blue: DAPI, red: FISH. (**K**) Serum SOD levels, n = 6 per group. (**L**,**M**) Representative ROS/DAPI staining images and quantification in colonic sections. Scale bar: 20 μm, n = 6 per group. Blue: DAPI, red: ROS. (ns, *p* > 0.05; * *p* < 0.05; ** *p* < 0.01; *** *p* < 0.001).

In addition, colonic epithelial PPARγ silencing abrogated PLA-mediated restoration of epithelial morphology, tight junction protein expression (ZO-1, OCLN), and barrier function, as reflected by persistently elevated serum LPS levels and bacterial translocation (Figure 5A–E). Furthermore, PPARγ was indispensable for PLA’s ability to maintain the goblet cell population (Figure 5F), the effects of PLA-on colonic *IL-6*, *IL-4* and *IL-10*, bacterial translocation and oxidative stress were abolished by PPARγ silencing (Figure 5H–M). Collectively, these data establish colonic epithelial PPARγ play a pivotal role in protecting against HTG-AP.

### 3.5. Pharmacological Activation of PPARγ with RSG Recapitulates the Protective Effects of PLA

Given the critical role of colonic epithelial PPARγ in HTG-AP severity, we next evaluated whether pharmacological activation of PPARγ with the agonist RSG could recapitulate the protective effects of PLA on intestinal barrier function and disease outcome (Figure S3).

**Figure 6:**
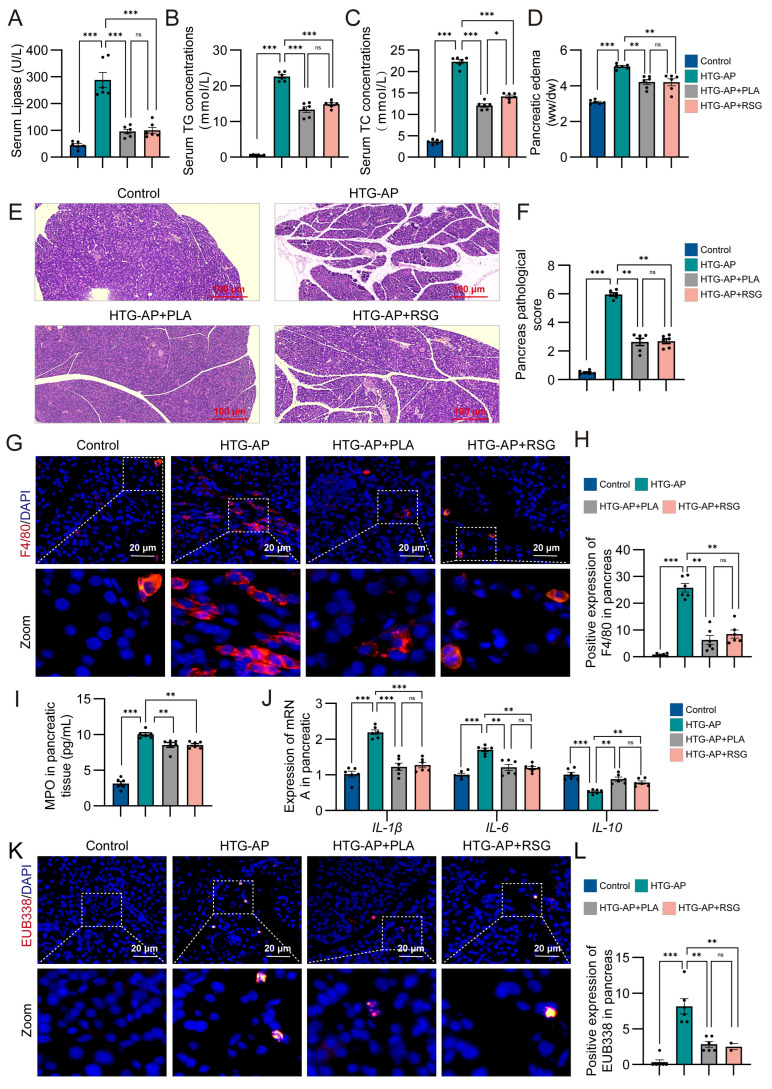
Pharmacological activation of PPARγ with RSG recapitulates the protective effects of PLA. (**A**) Serum LIP levels, n = 6 per group. (**B**) Serum TG levels, n = 6 per group. (**C**) Serum TC levels, n = 6 per group. (**D**) Pancreatic wet-to-dry weight ratio, n = 6 per group. (**E**,**F**) Representative pancreatic HE staining and histological score statistics. Scale bar: 100 μm, n = 6 per group. (**G**,**H**) Immunofluorescence staining and quantification of F4/80 (a macrophage marker) in pancreas. Scale bar: 20 μm, n = 6 per group. Blue: DAPI, red: F/480. (**I**) Pancreatic MPO levels determined by ELISA, n = 6 per group. (**J**) Pancreatic mRNA expression levels of *IL-1β*, *IL-6* and *IL-10*, n = 6 per group. (**K**,**L**) Representative EUB338/DAPI staining and quantification in pancreatic sections. Scale bar: 20 μm, n = 6 per group. Blue: DAPI, red: EUB338. (ns, *p* > 0.05; * *p* < 0.05; ** *p* < 0.01; *** *p* < 0.001).

RSG treatment markedly reduced pancreatic injury in HTG-AP mice, as evidenced by alleviated lipid metabolic disturbances (TG, TC), reduced pancreatic edema, and improved histological scores (Figure 6A–F). Furthermore, RSG suppressed pancreatic macrophage infiltration, MPO expression, and pro-inflammatory cytokine production, and reduced bacterial translocation into pancreatic tissues (Figure 6G–L).

**Figure 7:**
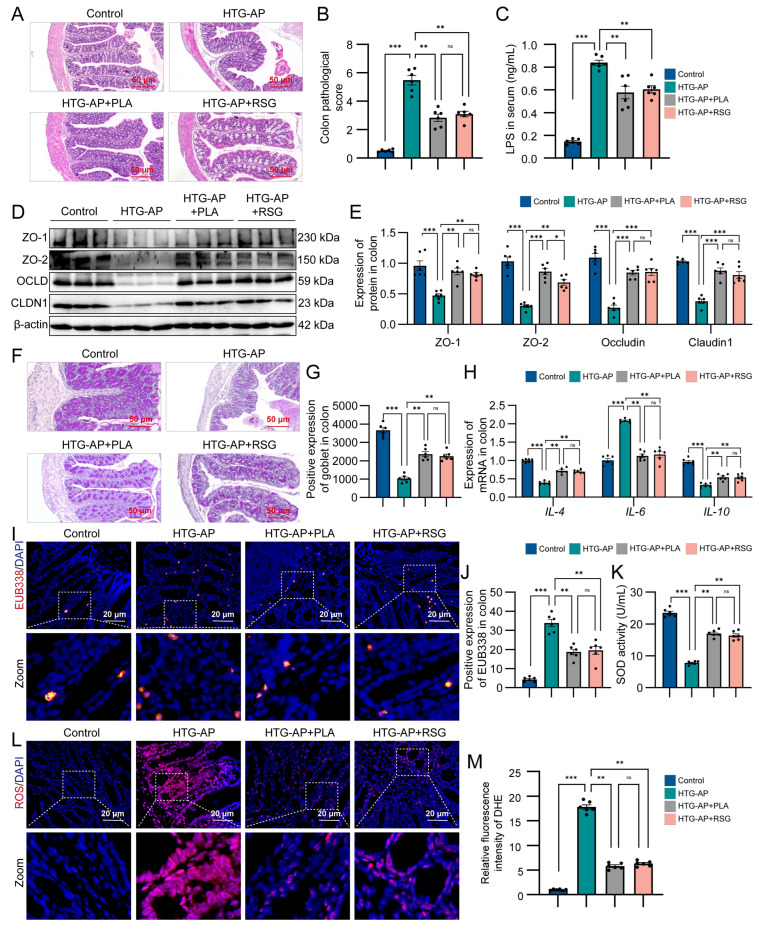
Pharmacological activation of PPARγ with RSG recapitulates the protective effects of PLA against colonic epithelial injury in HTG-AP mice. (**A**,**B**) Representative colonic HE staining images and histological scores. Scale bar: 50 μm, n = 6 per group. (**C**) Serum LPS levels, n = 6 per group. (**D**,**E**) WB analysis of tight junction proteins ZO-1, ZO-2, OCLN, and CLDN1 in colon, with quantification of their relative expression levels; β-actin served as the internal reference, n = 6 per group. (**F**,**G**) Representative PAS staining of colonic goblet cells and statistical analysis. Scale bar: 50 μm, n = 6 per group. (**H**) Colonic mRNA expression levels of *IL-4*, *IL-6* and *IL-10*, n = 6 per group. (**I**,**J**) Representative EUB338/DAPI staining images and quantification in colonic sections. Scale bar: 20 μm, n = 6 per group. Blue: DAPI, red: EUB338. (**K**) Serum SOD levels, n = 6 per group. (**L**,**M**) Representative ROS/DAPI staining images and quantification in colonic sections. Scale bar: 20 μm, n = 6 per group. Blue: DAPI, red: ROS. (ns, *p* > 0.05; * *p* < 0.05; ** *p* < 0.01; *** *p* < 0.001).

Consistent with its effects on the pancreas, RSG treatment also restored colonic epithelial integrity. This was evidenced by improved colonic histopathology, restored tight junction protein expression (ZO-1, OCLN), normalized goblet cell populations, and corrected inflammatory cytokine imbalance (*IL-6*, *IL-4*, *IL-10*). These improvements were accompanied by reduced serum LPS levels, decreased bacterial translocation and anti-oxidative stress (Figure 7A–M).

Collectively, these results demonstrate that pharmacological activation of PPARγ with RSG phenocopies the protective effects of PLA, further supporting colonic epithelial PPARγ as a central mediator of gut–pancreas axis homeostasis in HTG-AP.

## 4. Discussion

The pathogenesis of AP is complex and multifactorial [7,37,38]. As a highly aggressive subtype of AP, HTG-AP is clinically distinguished from other etiological forms by markedly aggravated pathological progression and unfavorable long-term prognosis with inferior overall clinical outcomes [39]. Mechanistically, the unique pathological deterioration of HTG-AP stems from the combined pathogenic effects of sustained lipotoxic injury [3,40,41] and disrupted gut-pancreas axis homeostasis [3,15], where these two pivotal pathological alterations mutually exacerbate local pancreatic inflammation and systemic metabolic disturbance, thereby further accelerating disease progression. In this study, we systematically investigated the protective effects and underlying molecular mechanisms of PLA in HTG-AP. Our results showed that endogenously derived metabolite PLA levels were significantly decreased in both HTG-AP patients and mice. Exogenous administration of PLA dose-dependently ameliorated biochemical abnormalities, pancreatic tissue injury, and inflammatory responses in HTG-AP mice. Dysfunction of the gut-pancreas axis is a key driver of HTG-AP progression, in which intestinal microbiota dysbiosis [42] and intestinal barrier disruption [43] aggravate pancreatic damage via bacterial translocation, systemic inflammation [44,45], and lipid metabolic disorders [20]. As core metabolic products of beneficial intestinal bacteria [46], SCFAs play a crucial regulatory role in the pathological progression of AP [47]. A reduction in SCFA levels further impairs intestinal barrier function [48]. Based on published evidence and phenotypic observations in this study, decreased SCFA levels are closely correlated with intestinal barrier dysfunction and exacerbated systemic inflammation, which may contribute to a potential vicious cycle of “gut dysbiosis–SCFA deficiency–pancreatic injury”. Specifically, the imbalanced gut microbiota, characterized by increased abundance of pathogenic bacteria and decreased abundance of beneficial bacteria, promotes the production of pro-inflammatory metabolites via the toll-like receptor 4-dependent pathway, thereby further exacerbating pancreatic damage [20]. In line with our data, PLA treatment obviously ameliorated colonic pathological damage, preserved intestinal epithelial barrier integrity. Meanwhile, PLA effectively modulated the gut microbial balance in HTG-AP mice, directionally promoting the proliferation of beneficial bacteria (e.g., *Alloprevotella*, *Bacteroides*) while suppressing the excessive proliferation of potential pathogenic bacteria such as *Escherichia-Shigella*. In this study, we objectively verified a significant correlation between gut microbiota dysbiosis and altered endogenous PLA levels under HTG-AP pathological conditions. Notably, our current experimental data are insufficient to support causal or regulatory relationships between intestinal flora variation and PLA metabolic changes. To further clarify the interplay among the above three factors, we will conduct in-depth mechanistic investigations in follow-up work via fecal microbiota transplantation, antibiotic-mediated microbiota depletion, and targeted bacterial colonization assays to supplement solid causal verification evidence.

Oxidative stress is a key upstream trigger of intestinal barrier impairment, as excessive ROS disrupt intestinal epithelial tight junction integrity and induce epithelial cell damage, forming a vicious cycle between oxidative injury and barrier dysfunction—consistent with our findings in HTG-AP [49,50]. Based on this, accumulating evidence has highlighted the regulatory role of microbiota-derived metabolites in modulating oxidative stress and maintaining cellular homeostasis; notably, PLA is a microbiota-derived metabolite, has been reported to enhance stress resilience by regulating oxidative stress-related pathways [51], which provides a theoretical basis for our observation that PLA mitigates HTG-AP-induced intestinal oxidative stress and barrier dysfunction.

PPARγ is a pivotal nuclear receptor that critically governs intestinal barrier function [52], intestinal inflammation [53,54,55], gut microbiota homeostasis [56] and lipid metabolism [57,58]. Dysregulation of PPARγ signaling is closely implicated in the pathogenesis of inflammatory bowel disease, eliciting intestinal mucosal damage, excessive inflammatory responses, oxidative stress and endoplasmic reticulum stress [53,59], thereby positioning targeted modulation of PPARγ as a promising therapeutic strategy for intestinal and metabolic disorders. In line with this, our mechanistic analyses using colonic RNA-seq and immunohistochemistry further demonstrated that the PPAR signaling pathway constituted the core dysregulated pathway in HTG-AP, alongside a notably reduced expression of colonic epithelial PPARγ in HTG-AP mouse models. Selective silencing of colonic epithelial PPARγ via AAV-shRNA almost completely abolished all protective effects of PLA, confirming that colonic epithelial PPARγ is an indispensable molecular target for PLA-mediated protection against HTG-AP. Previous studies have confirmed that RSG-a PPARγ agonist, significantly attenuates cerulein-induced AP [29]. Its protective effects are closely linked to PPARγ activation, inhibition of inflammatory signaling pathways, and alleviation of pancreatic tissue damage, providing critical pharmacological evidence for PPARγ as a therapeutic target in acute pancreatitis. The present study further validates the pivotal protective role of PPARγ in HTG-AP. Moreover, we demonstrated that PLA, a gut microbiota-derived metabolite, also exerts protective effects by targeting PPARγ. The villin promoter was used for intestinal epithelial targeting. However, without colocalization verification, off-target transduction into lamina propria immune cells cannot be excluded. Although further cell-specific validation is required in future studies, our functional results combined with the epithelial-targeted AAV system still support that epithelial PPARγ mediates the protective effects of PLA. In this study, RSG was applied solely as a positive control to verify the efficacy of PPARγ pathway activation and assist the functional validation of PLA. However, this study did not systematically characterize the systemic effects and toxicological safety profiles of PLA and RSG. Considering the non-specificity and systemic complexity of RSG-driven PPARγ activation, along with the absence of comprehensive safety evidence for PLA, we intend to address these research gaps in our future investigations. Collectively, our findings verify that PLA functions as a crucial protective metabolite targeting intestinal PPARγ to ameliorate gut barrier injury and HTG-AP inflammation, providing novel experimental evidence for PPARγ-centered intervention strategies for HTG-AP.

The core findings of this study are as follows: First, this work first validates the intrinsic association between endogenously derived metabolite PLA and HTG-AP pathogenesis, and demonstrates that exogenous PLA rescues HTG-AP-associated pancreatic biochemical perturbations and pathological lesions, complementing the unexplored biological correlation between PLA and HTG-AP. Previous studies have only characterized the anti-inflammatory and lipid-regulating properties of PLA, without investigating its biological relevance to HTG-AP [25,28,60]. Second, this study innovatively demonstrates that PLA modulates the gut–pancreas axis and enriches the gut microbiota-related regulatory mechanisms of HTG-AP and address the limitation of single-indicator assessment in existing studies [15]. Furthermore, this study linked PLA to the PPARγ signaling pathway. In addition, this study identifies an association between PLA and the PPARγ signaling pathway, which clarifies and refines the molecular mechanism underlying endogenous metabolite PLA-mediated regulation of HTG-AP progression.

This study has several limitations. First, the clinical sample size was relatively small (15 HTG-AP patients), which may compromise the generalizability and representativeness of our findings. In addition, owing to the retrospective design and long time interval of sample collection, detailed individual clinical data—including demographics, BMI, triglyceride levels, disease severity and medication history—were unavailable. Preclinical animal models cannot fully recapitulate the complex pathogenesis, individual heterogeneity and comorbidities of human HTG-AP, thereby limiting the clinical extrapolation and translational potential of the observed protective effects of PLA. Moreover, standardized administration regimens and systematic safety evaluation criteria for PLA remain lacking. Notably, only male animals were used in this study. The results may not be directly applicable to females, and sex-dependent differences in PLA sensitivity and PPARγ signaling cannot be excluded, further restricting clinical translation. Future large-sample, multicenter prospective clinical trials are required to validate the efficacy and safety of PLA in HTG-AP patients. Further studies should optimize clinical dosage and treatment regimens, evaluate long-term biosafety, and screen suitable populations for targeted intervention, so as to promote the clinical translation and application of PLA as a novel adjuvant therapy for HTG-AP. Second, the precise molecular pathway by which PLA activates colonic epithelial PPARγ and the detailed molecular network underlying PLA-mediated regulation of the gut microbiota remain to be fully elucidated. Third, the therapeutic efficacy of PLA was only validated in animal models, lacking clinical intervention trials. Therefore, its safety, efficacy, optimal dosage in humans, and potential effects of long-term administration have not been fully determined Notably, this study verified that dysregulated PLA metabolism serves as a distinct metabolic signature tightly correlated with HTG-AP pathological progression. Future work will conduct microbial knockout and rescue assays to validate microbiome-governed PLA metabolic modulation, elucidate the causal microbiome-PLA-HTG-AP regulatory cascade, and further dissect the molecular mechanisms underlying the protective effects of PLA against HTG-AP. In addition, commercial ELISA kits were used to detect serum LPS in this study, where lipid components in HTG serum may interfere with assay signals and impair LPS quantification accuracy. Meanwhile, housekeeping proteins including β-actin may vary under inflammatory conditions, limiting the validity of conventional normalization. Future work will adopt optimized lipid-removal pretreatment combined with LAL assay validation for LPS detection, and apply Ponceau S staining or stain-free imaging to enhance the overall reliability of protein quantification. And this study confirmed the basic analytical performance of the established LC-MS method but lacked full methodological validation including calibration curves, recovery, matrix effects and precision/accuracy assessment, which will be supplemented in our future work.

## 5. Conclusions

In summary, this study demonstrates that gut dysbiosis in HTG-AP decreases the abundance of taxa previously associated with PLA production and endogenously derived metabolite PLA levels, which further impairs PPARγ activation and disrupts intestinal barrier function. This perturbation triggers bacterial translocation, LPS-induced inflammation, oxidative stress, and pancreatic injury via the gut-pancreas axis. Notably, exogenous PLA supplementation can activate PPARγ, repair the intestinal barrier, and alleviate HTG-AP, thereby representing a promising therapeutic strategy for this disease.

## Data Availability

The original contributions presented in this study are included in the article/Supplementary Material. Further inquiries can be directed to the corresponding authors.

## Supplementary Materials

The following supporting information can be downloaded at: https://www.mdpi.com/article/10.3390/antiox15060676/s1, Figure S1: Animal experimental grouping and timeline (including PLA dose gradient); Figure S2: AAV intervention experimental design and verification of PPARγ expression; Figure S3: Animal experimental grouping and timeline; Table S1: Key reagents; Table S2: Primer information for RT-qPCR.

## Abbreviations

The following abbreviations are used in this manuscript:

AAV	Adeno-associated virus
AP	Acute pancreatitis
BCA	Bicinchoninicacid
cDNA	Complementary DNA
CER	Caerulein
CLDN1	Claudin 1
DAPI	4′,6 diamidino 2 phenylindole
ELISA	Enzyme linked immunosorbent assay
FISH	Fluorescence in situ hybridization
HE	Hematoxylin eosin staining
HTG	Hypertriglyceridemia
HTG-AP	Hypertriglyceridemic acute pancreatitis
LBD	Ligand-binding domain
LC-MS/MS	Liquid chromatography tandem mass spectrometry
LEfSe	Linear discriminant analysis effect size
LIP	Lipase
LPS	Lipopolysaccharide
MPO	Myeloperoxidase
OCLN	Occludin
P-407	Poloxamer 407
PAS	Periodic acid Schiff staining
PCoA	Principal coordinate analysis
PBS	Phosphate buffer saline
PLA	Phenyllactic acid
PPAR	Peroxisome proliferator-activated receptor
qRT PCR	Quantitative real time polymerase chain reaction
RNA-seq	RNA sequencing
ROS	Reactive oxygen species
SCFA	Short chain fatty acid
SEM	Standard error of the mean
SOD	Superoxide dismutase
TC	Total cholesterol
TG	Triglyceride
ZO-1	Zonula occludens-1
ZO-2	Zonula occludens-2
