# Supplementary material for: Phenyllactic Acid Restores Intestinal Epithelial Barrier to Alleviate Hypertriglyceridemic Acute Pancreatitis via a PPARγ-Dependent Mechanism
Source: Antioxidants (Basel). 2026 May 28;15(6):676. doi: 10.3390/antiox15060676 (PMC13295703; doi:10.3390/antiox15060676)
Supplement: Supplementary file 1 [file antioxidants-15-00676-s001.zip › antioxidants-4267905-supplementary.pdf]

Supplementary Materials

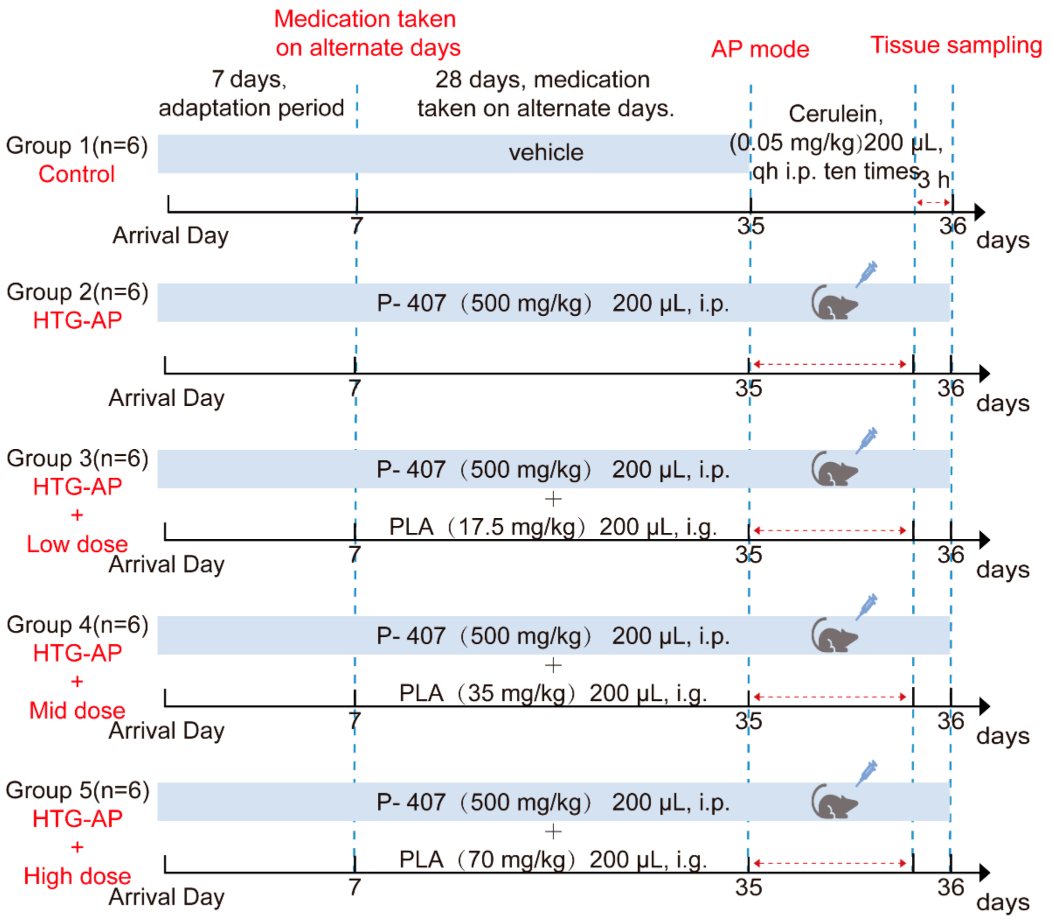

Figure S1, related to **Figure 1**. Animal experimental grouping and timeline (including PLA dose gradient). Male mice (n=6 per group) were randomized into 5 groups for a 36-day experiment.

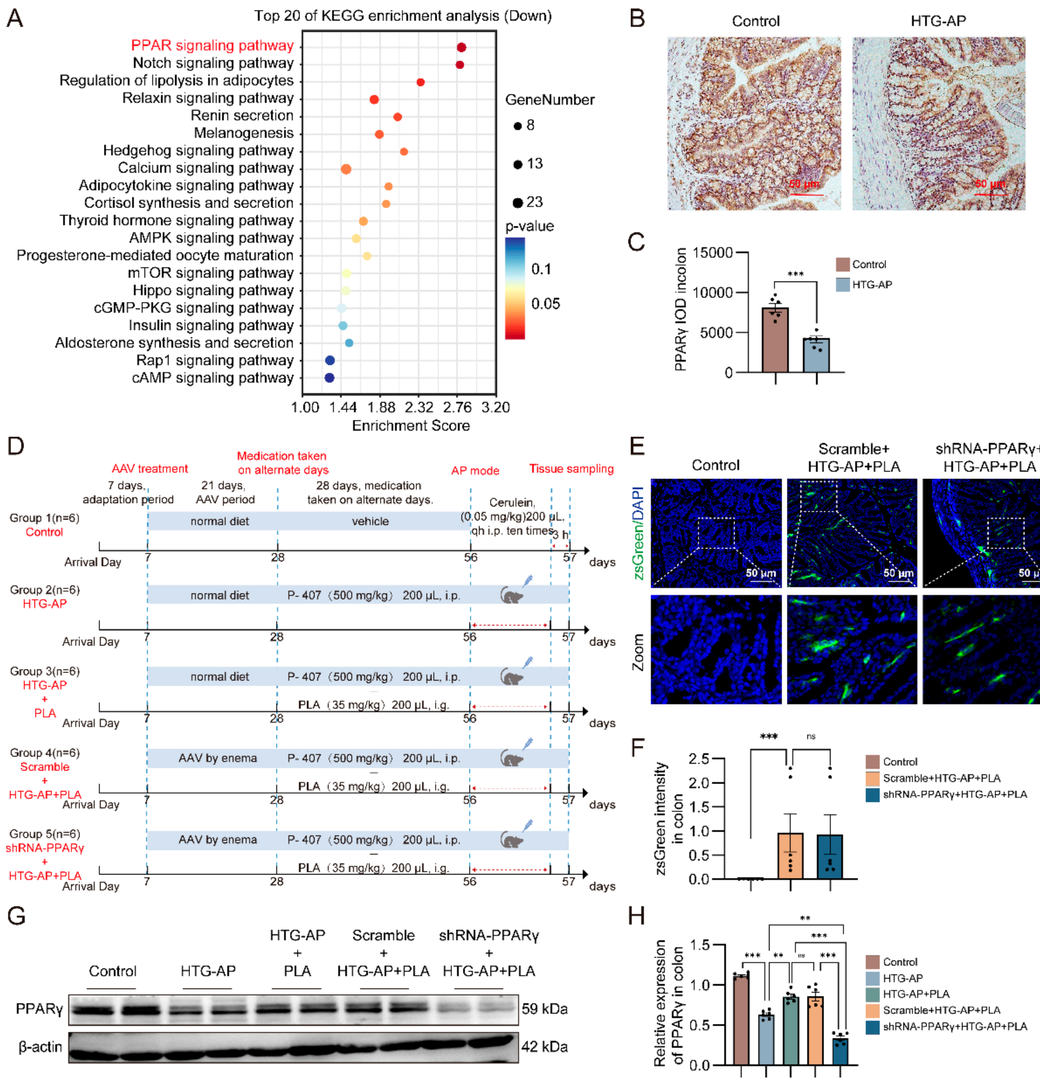

Figure S2, related to **Figure 4**. AAV intervention experimental design and verification of PPARγ expression. (A) KEGG enrichment analysis of downregulated pathways in HTG-AP, highlighting the PPAR signaling pathway as the most significantly enriched. The size of the dots represents the number of genes, and the color gradient represents the p-value. (B-C) Representative immunohistochemical staining of PPARγ in colonic tissues from control and HTG-AP mice. Scale bar: 50 μm, n=6 per group. (D) Animal experimental grouping and timeline. Male mice (n=6 per group) were randomised into 5 groups for a 57-day experiment. (E-F) Representative zsGreen/DAPI staining images of colon tissue sections and statistical analysis. Scale bar: 50 μm, n = 6 per group. Blue: DAPI, green: zsGreen. (G-H) WB analysis of PPARγ in colon, with statistical analysis of its relative expression levels, β-actin served as the internal reference, n=6 per group. *p<0.05, **p<0.01, ***p<0.001.

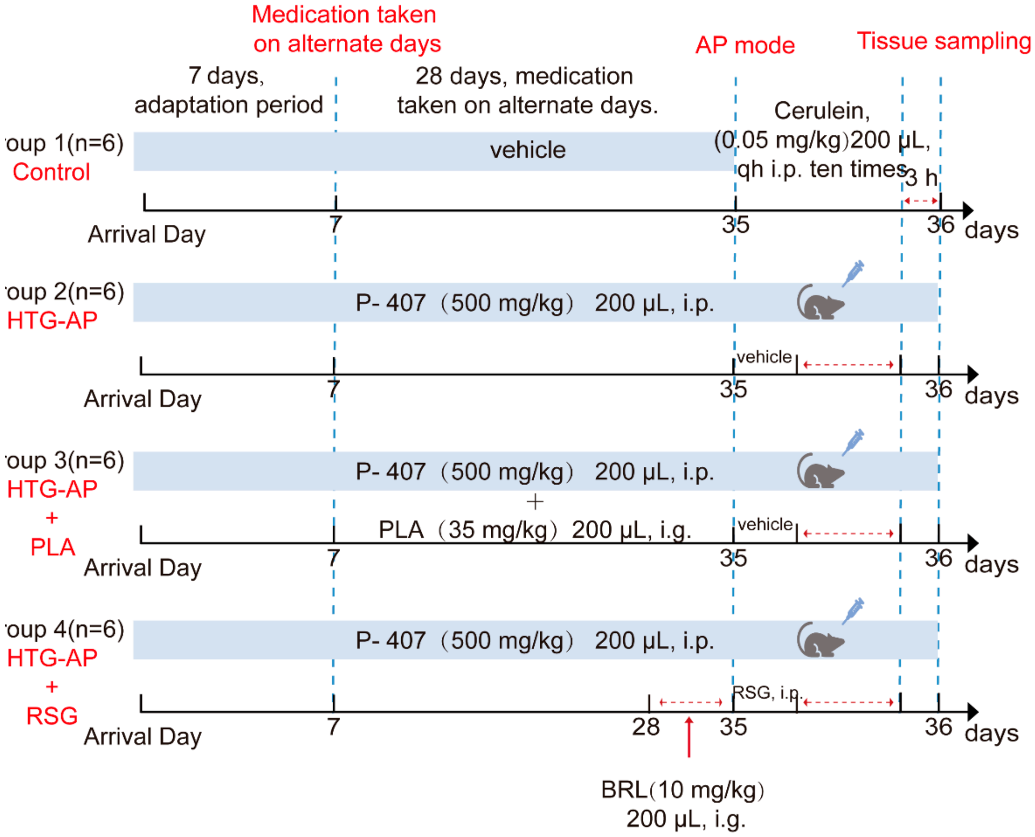

Figure S3, related to **Figure 6.** Animal experimental grouping and timeline. Male mice (n=6 per group) were randomized into 4 groups for a 36-day experiment.

Table S1:

Key reagents.

REAGENT	SOURCE	IDENTIFIER
P-407	Aladdin	Cat#: P4344419
cerulein	Aladdin	Cat#: C305198
D-(+)-3-Phenyllactic acid	Aladdin	Cat#: P102443
Rosiglitazone	Topscience	Cat#: 122320-73-4
DL-3-Phenyllactic acid	MedChemExpress	Cat#: HY-W017162R
DL-3-Phenyllactic acid-d3	MedChemExpress	Cat#: HY-W017162S
Acetonitrile	Damas-beta	CAS: 75-05-8
Cy3-conjugated Goat anti-Rabbit IgG (H+L)	ABclonal	Cat#: AS007
Cy3-conjugated Goat anti-Mouse IgG (H+L)	ABclonal	Cat#: AS008

Table S2:

Primer information for RT-qPCR.

Gene	Forward primer	Reverse primer
IL-1β	GTGGCTGTGGAGAAGCTGTG	GAAGGTCCACGGGAAAGACAC
IL-4	AGATCATCGGCATTTTGAACG	TTTGGCACATCCATCTCCG
IL-6	TAGTCCTTCCTACCCCAATTTCC	TTGGTCCTTAGCCACTCCTTC
IL-10	TCACTCTTCACCTGCTCCAC	CTATGCTGCCTGCTCTTACTC
β-actin	GGCTGTATTCCCCTCCATCG	CCAGTTGGTAACAATGCCATGT
